# 1335. Etiological and Epidemiological Features of Acute Meningitis and Encephalitis in Hospitalized Patients, Kazakhstan, 2019 - 2020

**DOI:** 10.1093/ofid/ofad500.1172

**Published:** 2023-11-27

**Authors:** Bakhytkul Zhakipbayeva, Dmitriy V Berezovskiy, Yekaterina V Bumburidi, Nursulu A Berdiyarova, Kaldygul D Kulzhanova, Murat A Agabayev, Toni Whistler, Pawan Angra, Daniel A Singer, James J Sejvar

**Affiliations:** U.S. Centers for Disease Control and Prevention, Central Asia Regional Office, Almaty, Kazakhstan, Almaty, Almaty, Kazakhstan; U.S. Centers for Disease Control and Prevention, Central Asia Office, Almaty, Kazakhstan, Almaty, Almaty, Kazakhstan; CDC/Central Asia office, Almaty, Almaty, Kazakhstan; Shymkent City Infectious Disease Hospital, City Department of Health Care, Shymkent, Kazakhstan, Shymkent, Ongtustik Qazaqstan oblysy, Kazakhstan; Shymkent City Infectious Disease Hospital, City Department of Health Care, Shymkent, Kazakhstan, Shymkent, Ongtustik Qazaqstan oblysy, Kazakhstan; Shymkent City Infectious Disease Hospital, City Department of Health Care, Shymkent, Kazakhstan, Shymkent, Ongtustik Qazaqstan oblysy, Kazakhstan; U.S. Centers for Disease Control and Prevention, Atlanta, GA, United States of, Atlanta, Georgia; US CDC, Atlanta, Georgia; U.S. Centers for Disease Control and Prevention, Atlanta, GA, United States of America, Atlanta, Georgia; U.S. Centers for Disease Control and Prevention, Atlanta, GA, United States of America, Atlanta, Georgia

## Abstract

**Background:**

Acute meningitis and encephalitis (AME) are neurological inflammatory conditions associated with substantial morbidity and mortality caused by different pathogens. In Kazakhstan, comprehensive testing to identify causal pathogens is not routinely performed. We conducted syndromic surveillance for AME in the south region (fig.1), an area of Kazakhstan with a particularly high incidence.

**Figure 1.**
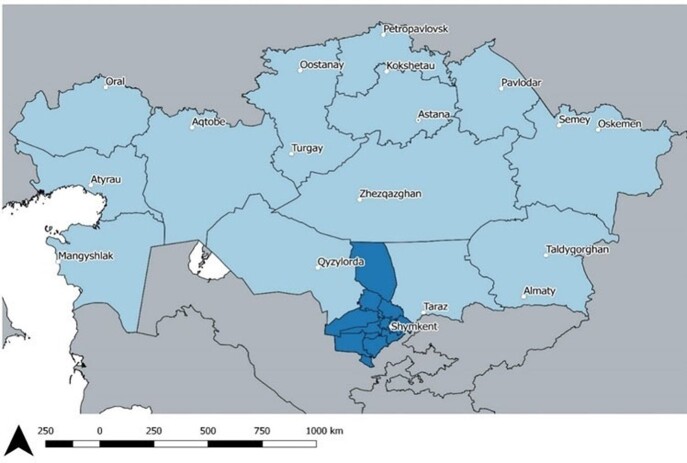
Location of acute meningitis and encephalitis study, Shymkent city and Turkestan Oblast, Kazakhstan, 2019-2020

The study locations are shown in dark blue. Population of Turkestan was 2.0 million and Shymkent was 1.0 million in 2019.

**Methods:**

We enrolled all patients hospitalized and meeting our AME case definition in the Shymkent City Infectious Diseases Hospital for 12 months beginning May 2019. We abstracted demographic, clinical, epidemiology and laboratory data, and collected cerebrospinal fluid (CSF) and whole blood to test for causative pathogens by cascading algorithm (fig.2). We performed serotyping or PCR to identify *Neisseria meningitidis* serogroups and sequencing of Enterovirus VP1 gene.

**Figure 2.**
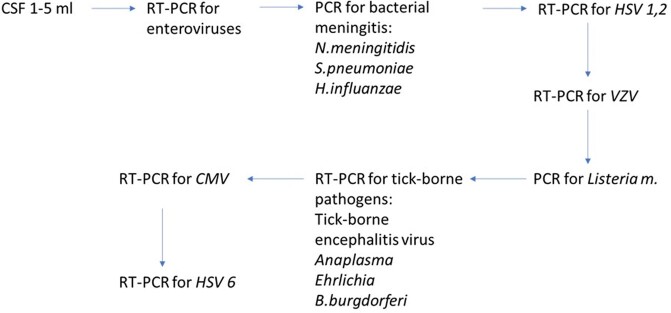
PCR testing for detection of causal pathogens by cascade algorithm

The arrows show the order in which the samples are tested; in case of positive result further testing stops.

**Results:**

Of 1344 subjects (median age 10 years, range 0.5 months - 72 years, male – 55%) with AME, the causal pathogen was identified in 84.3% (81.6% viral and 2.7% bacterial). Viral pathogens included enteroviruses (79%), herpes simplex virus 1 or 2 (0.8%), varicella-zoster virus (0.8%), tick-borne encephalitis (0.9%), and herpes simplex virus 6 (0.2%) (tab.1). Among 36 participants with bacterial infections, 61% had *N. meningitidis*, 36% *S. pneumoniae*, and 3% *H. influenzae*. Of 28 enterovirus samples sequenced, 54% were Coxsackievirus A6, A10; 46% were Echovirus E6. *N. meningitidis* serogroups A and W138 prevailed. Enteroviral AME peaked in summer (fig.3) and incidence rate (IR) was 2.5 times higher than in summer 2017, according to our previous study. IR was 0.7 per 100,000 population for meningococcal AME and 34.6 per 100,000 for enteroviral AME. Enteroviral AME incidence was highest among 7-14 year olds (fig.4).

**Table 1.**
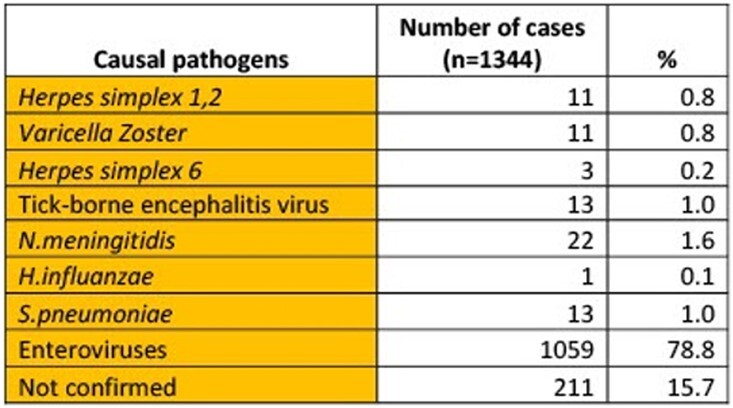
Etiology of acute meningitis and encephalitis cases (n=1344), Shymkent City and Turkestan Oblast, Kazakhstan, 2019-2020

In 1344 patients with AME, the causal pathogen was identified in 84.3% (81.6% viral and 2.7% bacterial) cases.

**Figure 3.**
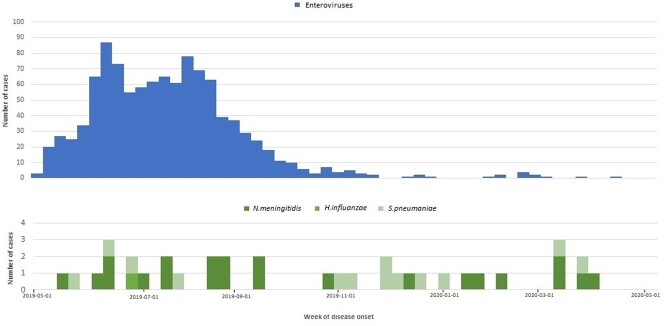
Epidemiological curves of laboratory-confirmed cases of acute encephalitis and meningitis, Shymkent City and Turkestan Oblast, Kazakhstan, 2019-2020

**Figure 4.**
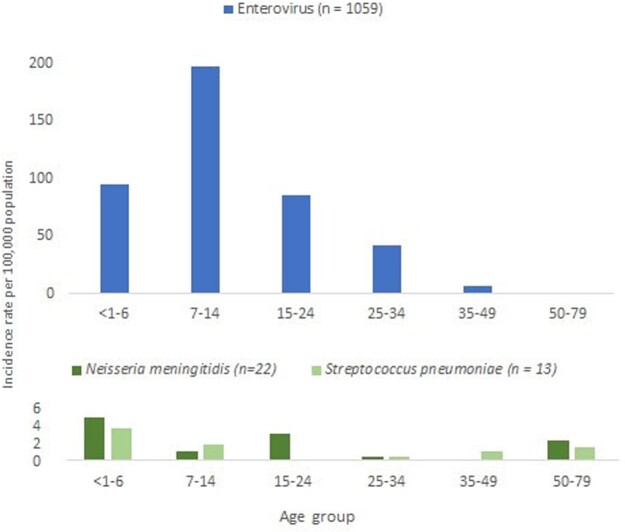
Incidence rates of encephalitis and meningitis by age group and etiology, Shymkent City and Turkestan Oblast, Kazakhstan, 2019-2020

**Conclusion:**

Viral pathogens, specifically enteroviruses, were the most common cause of AME. We found tick-borne encephalitis (TBE) for the first time in a region where TBE is not known to be endemic. CSF PCR had a higher yield than the culture method for identifying bacterial AME. Routine PCR for AME would improve pathogen identification and clinical care. Pathogens associated with AME should be added to national surveillance to help inform prevention and control strategies such as vaccination.

**Disclosures:**

**All Authors**: No reported disclosures

